# Author Correction: Diversity amongst human cortical pyramidal neurons revealed via their sag currents and frequency preferences

**DOI:** 10.1038/s41467-022-29220-9

**Published:** 2022-03-24

**Authors:** Homeira Moradi Chameh, Scott Rich, Lihua Wang, Fu-Der Chen, Liang Zhang, Peter L. Carlen, Shreejoy J. Tripathy, Taufik A. Valiante

**Affiliations:** 1 Krembil Brain Institute, University Health Network, Toronto, ON Canada; 2 Department of Electrical and Computer Engineering, University of Toronto, Toronto, ON Canada; 3 Max Planck Institute of Microstructure Physics, Halle, Germany; 4 Departments of Medicine & Physiology, University of Toronto, Toronto, ON Canada; 5 Institute of Biomedical Engineering, University of Toronto, Toronto, ON Canada; 6 Krembil Centre for Neuroinformatics, Centre for Addiction and Mental Health, Toronto, ON Canada; 7 Institute of Medical Sciences, University of Toronto, Toronto, ON Canada; 8 Department of Psychiatry, University of Toronto, Toronto, ON Canada; 9 Division of Neurosurgery, Department of Surgery, University of Toronto, Toronto, ON Canada

**Keywords:** Cellular neuroscience, Neurophysiology

Correction to: *Nature Communications* 10.1038/s41467-021-22741-9, published online 03 May 2021.

The original version of this Article contained errors in Fig. 3.

In Fig. 3f, data points corresponding to putative interneurons, which were placed in an additional figure, Supplementary Fig. 7, during the revision process, were inadvertently not subsequently removed from Fig. 3f as they should have been. These data points have been removed from the correct version of the figure.

In Fig. 3f and 3h, the groups labelled ‘layer 2&3’ and ‘layer 5’ contained some data points that were inadvertently duplicated during the preparation of the figures. The duplicated data points corresponded to 10 interneurons in the ‘layer 2&3’ group and 15 interneurons in the ‘layer 5’ group. The duplicated data points have been removed from the correct version of the figure.

This duplication error related to plotting of the data points only, and the statistical analyses, figure legends, and Source data for Fig. 3f and Fig. 3h were not affected.

In Fig. 3i, the groups labelled ‘Layer 2&3’, ‘Layer 3c’ and ‘Layer 5’ contained some data points that were inadvertently duplicated during the preparation of the figures. The duplicated data points corresponded to two interneurons in the ‘Layer 2&3’ group, one interneuron in the ‘layer 3c’ group, and three interneurons in the ‘Layer 5 group’. The duplicated data points have been removed from the correct version of the figure. This duplication error related to plotting of the data points only, and the statistical analyses and figure legend was not affected. However, the Source data for panel 3i was were inadvertently omitted from the Source Data File in the original version of the Article. The Source Data File has been updated to include the data from Fig. 3i.

In addition, the y axis of Fig. 3f incorrectly listed mV as unit, instead of ms. This has been corrected in the PDF and HTML version of the article.

The correct version of Fig. 3 is 
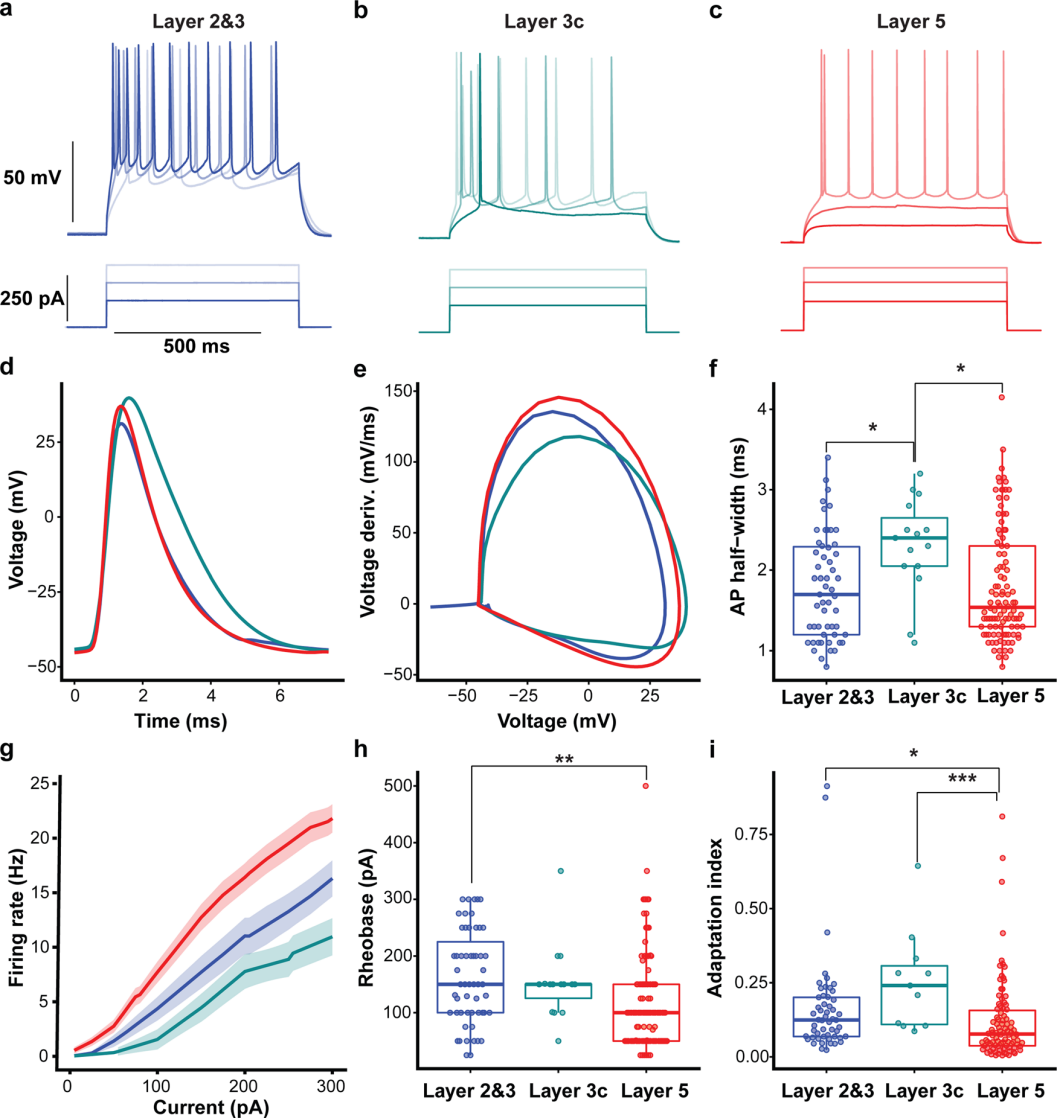


which replaces the previous incorrect version:
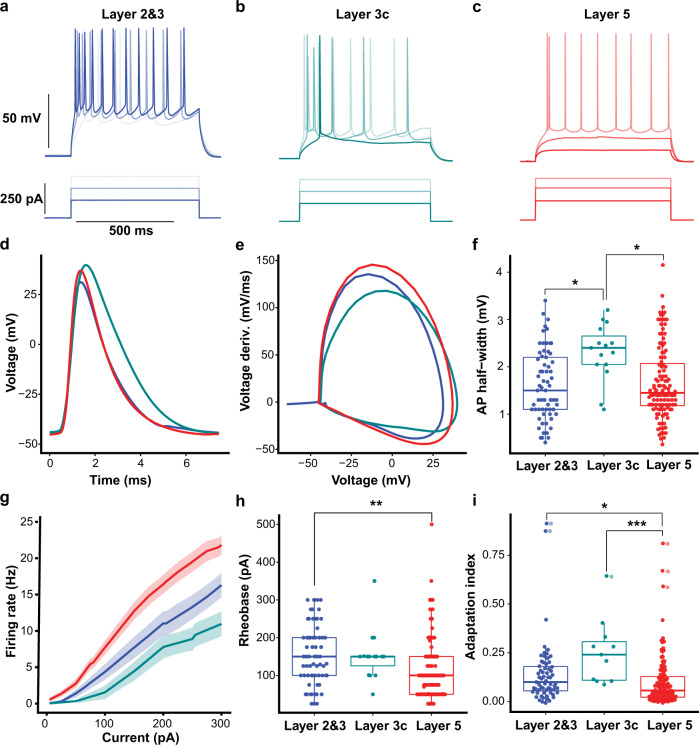


Furthermore, in the original version of this Article funding from ‘Kremblin Foundation’ was omitted from the Acknowledgements. This has been corrected in both the PDF and HTML versions of the Article.

## Supplementary information

Updated Source Data

