# Supplementary material for: Diversity amongst human cortical pyramidal neurons revealed via their sag currents and frequency preferences
Source: Nat Commun. 2021 May 3;12:2497. doi: 10.1038/s41467-021-22741-9 (PMC8093195; doi:10.1038/s41467-021-22741-9)
Supplement: Supplementary file 1 — Supplementary Information [file 41467_2021_22741_MOESM1_ESM.pdf]

Supplementary Information

Diversity amongst human cortical pyramidal neurons revealed via their sag currents and frequency preferences

Homeira Moradi Chameh[1], Scott Rich[1], Lihua Wang[1], Fu-Der Chen[2,3], Liang Zhang[1,4], Peter L. Carlen[1,4,5], Shreejoy J. Tripathy[6,7,8†], Taufik A. Valiante[1,2,5,7,9*†]

[1]Krembil Brain Institute, University Health Network, 60 Leonard Street, Toronto, ON, Canada, M5T2S8. [2]Department of Electrical and Computer Engineering, University of Toronto, 10 king's College Rd, Toronto, ON, Canada, M5S 3G8. [3]Max Planck Institute of Microstructure Physics, Weinberg 2, 06120, Halle, Germany. [4]Departments of Medicine & Physiology, University of Toronto, King's College Circle, Medical Science Building, Toronto, ON, Canada, M5S 1A8. [5]Institute of Biomedical Engineering, University of Toronto, Rosebrugh Building, 164 College Street, Toronto, ON, Canada, M5S 3G9. [6]Krembil Centre for Neuroinformatics, Centre for Addiction and Mental Health, 250 College Street, Toronto, ON, Canada, M5T 1R8. [7]Institute of Medical Sciences, University of Toronto, King's College Circle, Medical Science Building, Toronto, ON, Canada, M5S 1A8. [8]Department of Psychiatry, University of Toronto, 250 College Street, Toronto, ON, Canada, M5T 1R8. [9]Department of Surgery, Division of Neurosurgery, University of Toronto, Stewart Building, 149 College Street, Toronto, ON, Canada. M5T 1P5.

[†]These authors contributed equally.
* taufik.valiante@uhn.ca

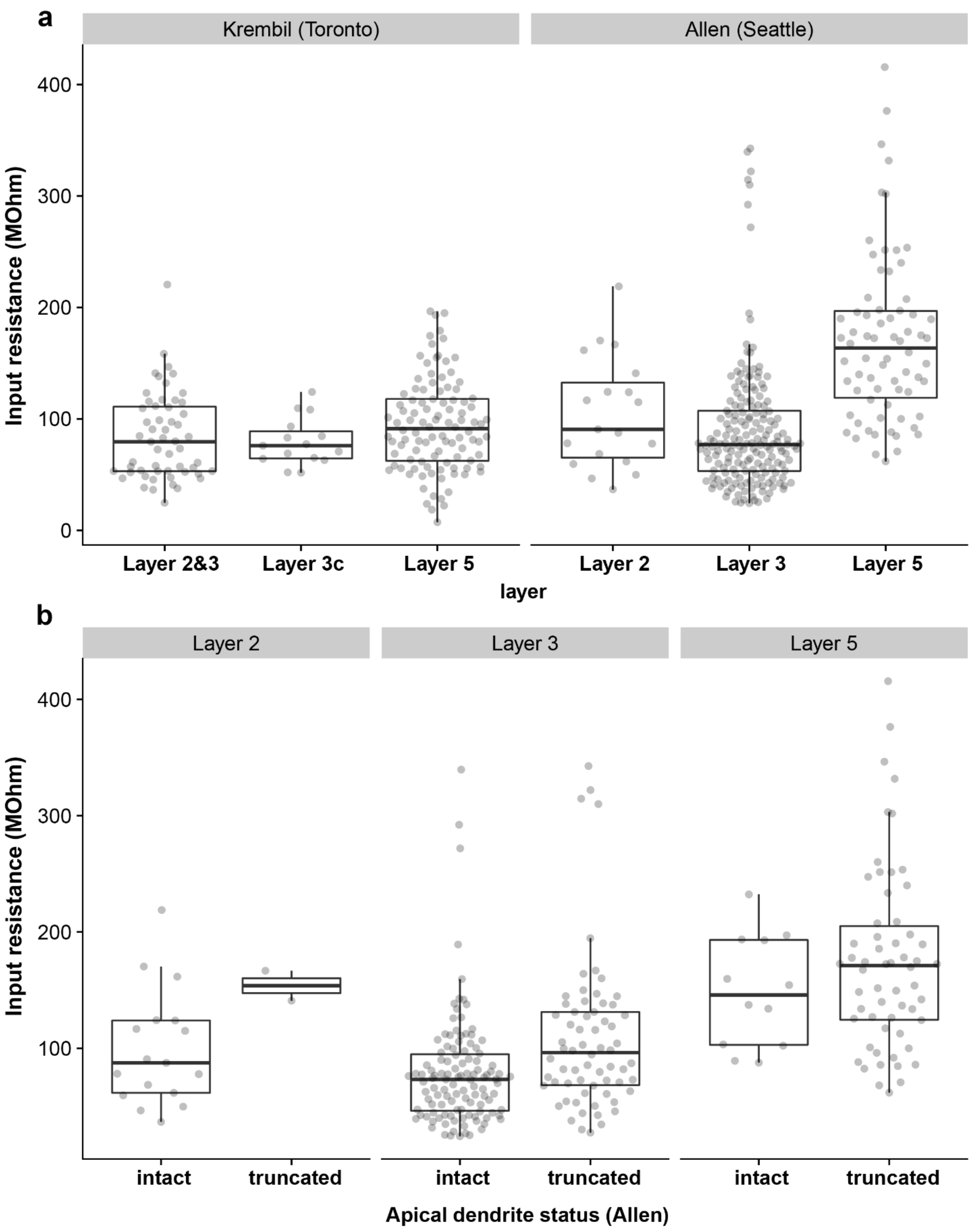
a
Krembil (Toronto)
Allen (Seattle)
Input resistance (MOhm)
400
300
200
100
0
Layer 2&3
Layer 3c
Layer 5
Layer 2
Layer 3
Layer 5
layer
b
Layer 2
Layer 3
Layer 5
Input resistance (MOhm)
400
300
200
100
intact
truncated
intact
truncated
intact
truncated
Apical dendrite status (Allen)

Supplementary Figure 1: Comparison of input resistance measurements in human cortical pyramidal cells collected from the Krembil Brain Institute and the Allen Institute. a Input resistance measurements from Krembil Brain Institute cohort (left, n = 56, 15, 105 for L2&3, L3c, L5, respectively) and Allen Institute cohort (right, n = 19, 181, 70 for L2, L3, L5, respectively). Note the trend of increased input resistances in L5 pyramidal cells relative to L2&3 neurons. **b** Same as right panel of a, but data have been grouped by whether the apical dendrite is intact or truncated (L2, n = 17 intact, 2 truncated; L3, 115 intact, 66 truncated; L5, 12 intact, 58 truncated). According to the Allen Institute's documentation, an apical dendrite is "intact" if the entire length of the primary dendrite was contained within the thickness of the slice and "truncated" if the primary dendritic branch was cut off at either slice surface. While dendrite truncation tends to increase input resistances, the overall relationship that input resistances in L2&3 are smaller than in L5 holds for neurons with confirmed intact primary dendrites. Boxplots denote interquartile range and whiskers denote data range excluding outliers. Source data are provided as a Source Data file.

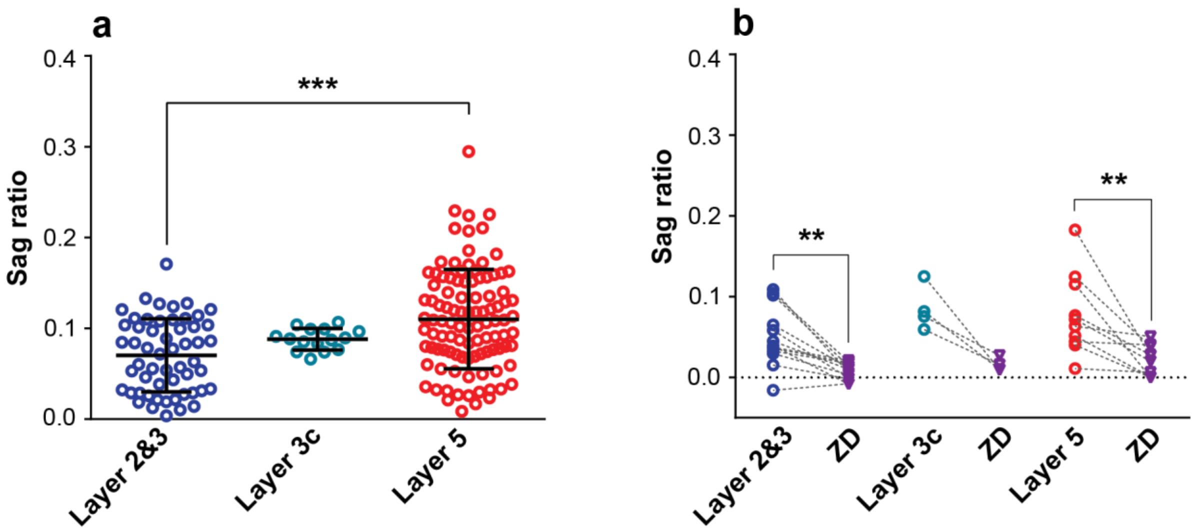

Supplementary Figure 2: Sag differences between cortical layers are robust to normalizing for input resistance differences using the dimensionless sag ratio measure. a Same as Fig 2b, but data are plotted using the sag ratio measure . L5 pyramidal cells had significantly larger sag ratio than L2&3 pyramidal cells ($p<0.0001$ between L2&3 and L5; One-Way ANOVA post hoc with Dunn's multiple comparison test. L2&3: 0.07 ± 0.04 mV, n=55; L3c: 0.08 ± 0.01 mV, n=15; L5: 0.11 ± 0.05 mV, n=103). Data presented as mean±SD **b** Same as Fig 2e, showing bath application of Ih blocker ZD7288 (10 µM) reducing sag ratio in pyramidal cells from both layers (L2&3-ZD: p=0.0005, n=13; L3c-ZD: p=0.1250, n=4; L5-ZD: p=0.0020, n=10; Wilcoxon matched-pairs signed rank test). Source data are provided as a Source Data file.

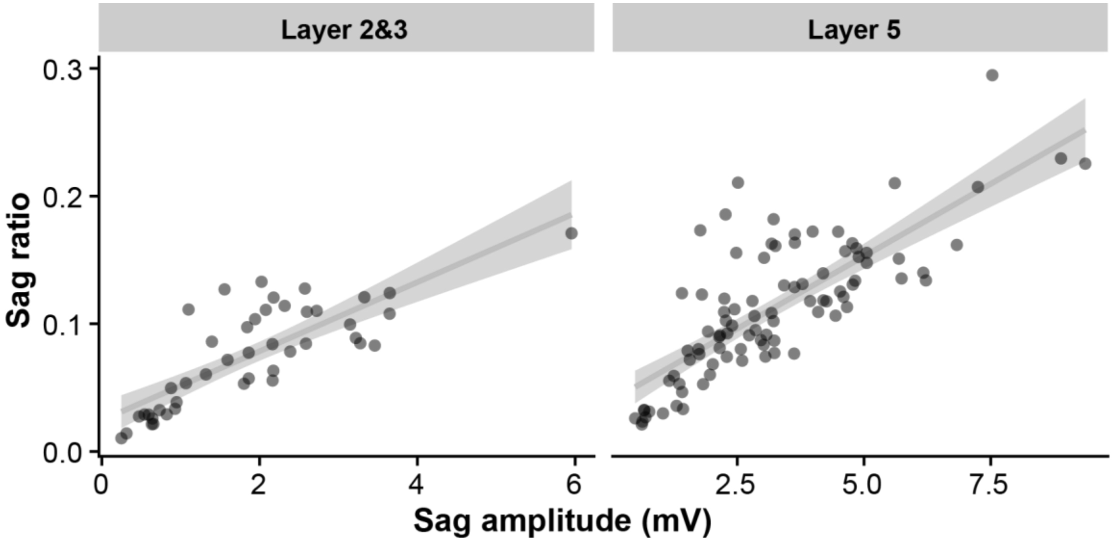

Supplementary Figure 3: Correlation between sag voltage and sag ratio in data from the Krembil Brain Institute cohort. Source data are provided as a Source Data file.

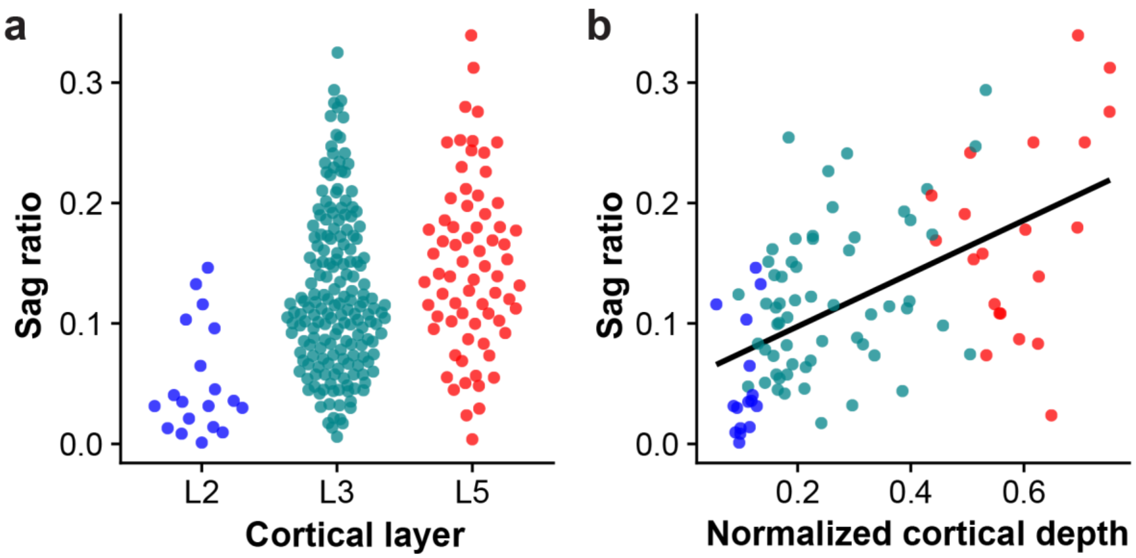

Supplementary Figure 4: Sag ratio measurements in human cortical pyramidal cells collected from the Allen Institute. **a** Sag ratio measurement collected in each cortical layer (n = 19, 181, 70 for L2, L3, L5, respectively). **b** Sag ratio as a function of normalized cortical depth from the pial surface (n = 16, 59, 21 for L2, L3, L5, respectively). Cells are a subset of those shown in (a) with normalized cortical depth information available. Line indicates best fit line. Source data are provided as a Source Data file.

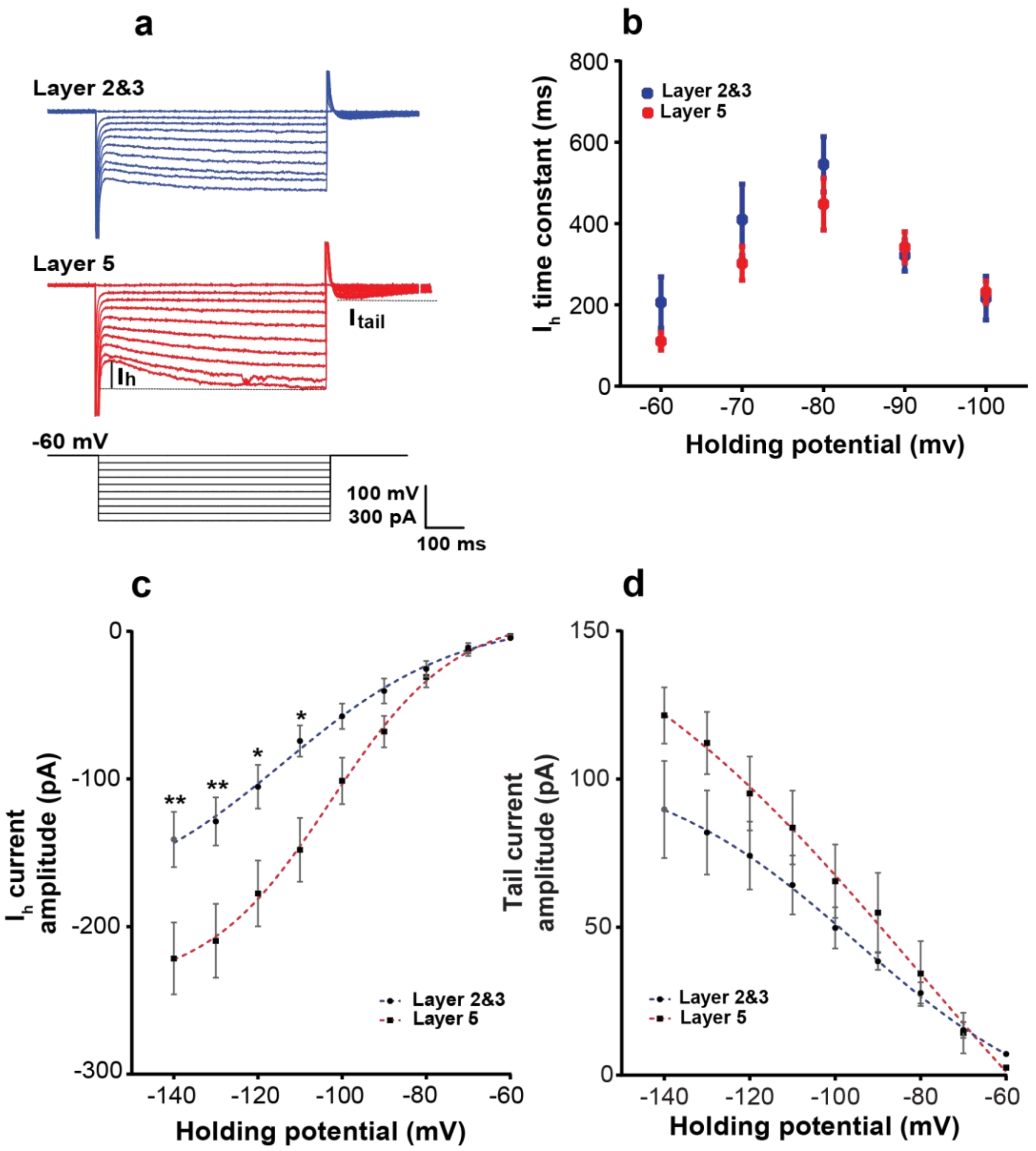

Supplementary Figure 5: The kinetics of I_h are similar between L2&3 and L5 pyramidal cells. a Example voltage-clamp recordings of L2&3 and L5 pyramidal cells. Annotations show calculation of I_h and I_{tail}. **b** Voltage clamp recordings of current show that I_h time constants are similar between L2&3 and L5 pyramidal cells ($p \geq 0.9999$;Two-Way ANOVA post hoc with Bonferroni's multiple comparison test, L2&3: n= 6, L5: n=10) . **c** L5 pyramidal cells had significantly larger I_h amplitude compared to L2&3 pyramidal cells (**p=0.0088, * p=0.0265;Two-Way ANOVA post hoc with Bonferroni's multiple comparison test). **d** Quantification of I_{tail} at the end of each holding potential revealed that there was no significant difference between L2&3 and L5 ($p \geq 0.9999$;Two-Way ANOVA post hoc with Bonferroni's multiple comparison test). The dashed lines in **c** and **d** indicates the fit to a Boltzmann function. These data suggest that the difference between sag voltages in L2&3 and L5 pyramidal cells are not due to differences in the kinetics of HCN channels. Data presented as mean ±SEM, L2&3: n= 6, L5: n=10). Source data are provided as a Source Data file.

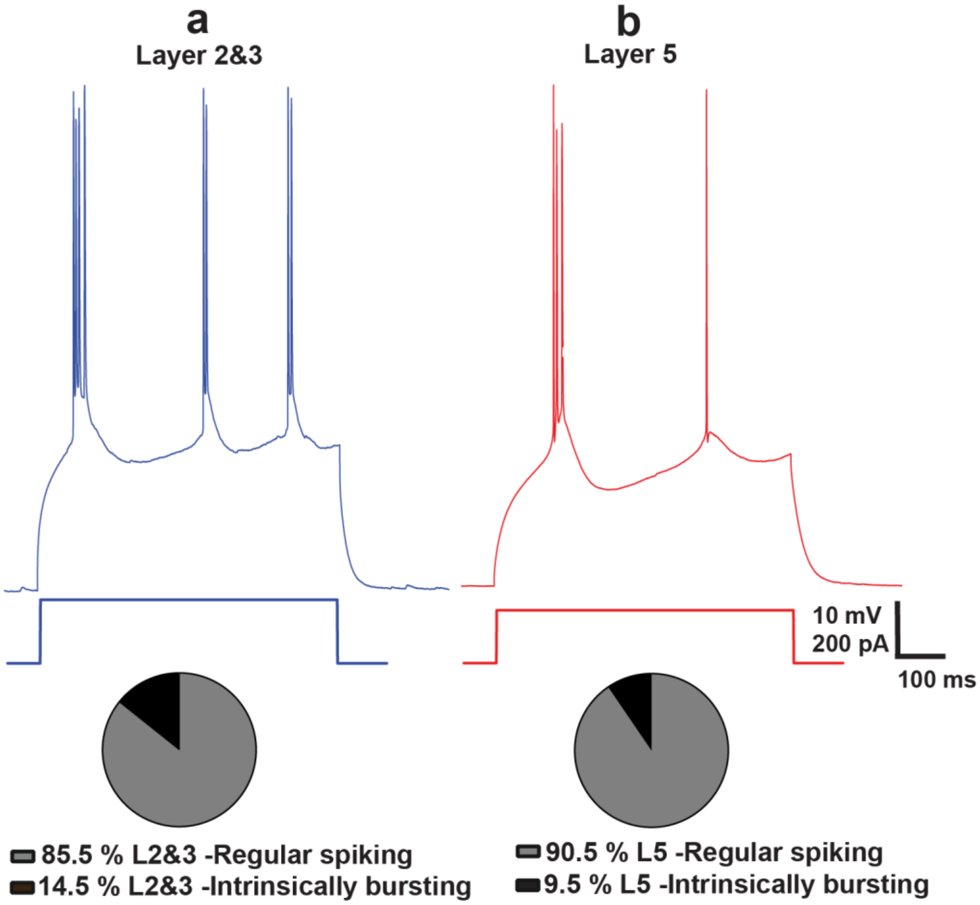

Supplementary Figure 6: Distribution of different firing patterns across L2&3 and L5 pyramidal cells. a,b Example voltage traces of intrinsically bursting neurons at rheobase. L2&3 (a) and L5 (b) pyramidal cells tend to have more regular spiking than intrinsically bursting neurons.The percentage of intrinsically bursting neurons recorded in L2&3 was slightly higher than L5. Source data are provided as a Source Data file.

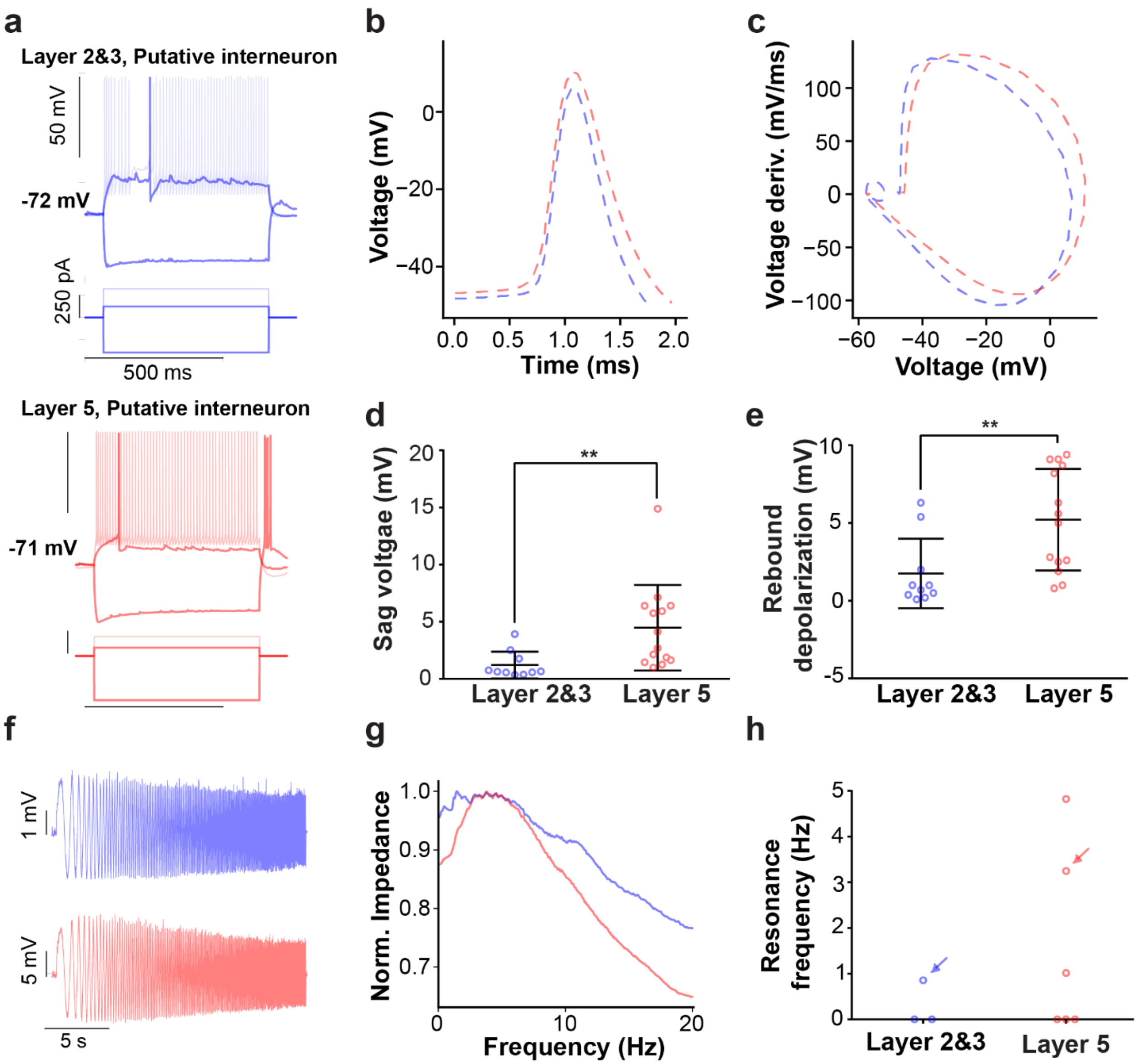

Supplementary Figure 7. Electrophysiological and subthreshold resonance properties of putative interneurons recorded in L2&3 and L5. a Example voltage responses and current steps for an example putative interneuron from L2&3 (top) and L5 (bottom). **b,c** Action potential waveform (b) and action potential phase plot (c) averaged over recorded putative interneurons in L2&3 (blue) and L5 (red). **d,e** Sag amplitude (p= 0.0012; two-sided t-test, Mann-Whitney, L2&3: 1.2 ± 1.1 mV, n=10; L5: 4.5± 3.7 mV, n=14) (d) and post-hyperpolarization rebound depolarization amplitude (p= 0.0032; two-sided t-test, Mann-Whitney, L2&3: 1.7 ± 2.2 mV, n=10; L5: 5.2± 3.2 mV, n=14) (e) reveals greater amounts of sag and rebound depolarization amplitude in L5 putative interneurons compared to L2&3. **f,g** Example subthreshold voltage responses (f) and normalized impedance (g) following ZAP current injection for example neurons shown in **a**. **h** Subthreshold resonance frequencies show a trend for more subthreshold resonance in L5. Arrows indicate neurons highlighted in **f**. Source data are provided as a Source Data file.

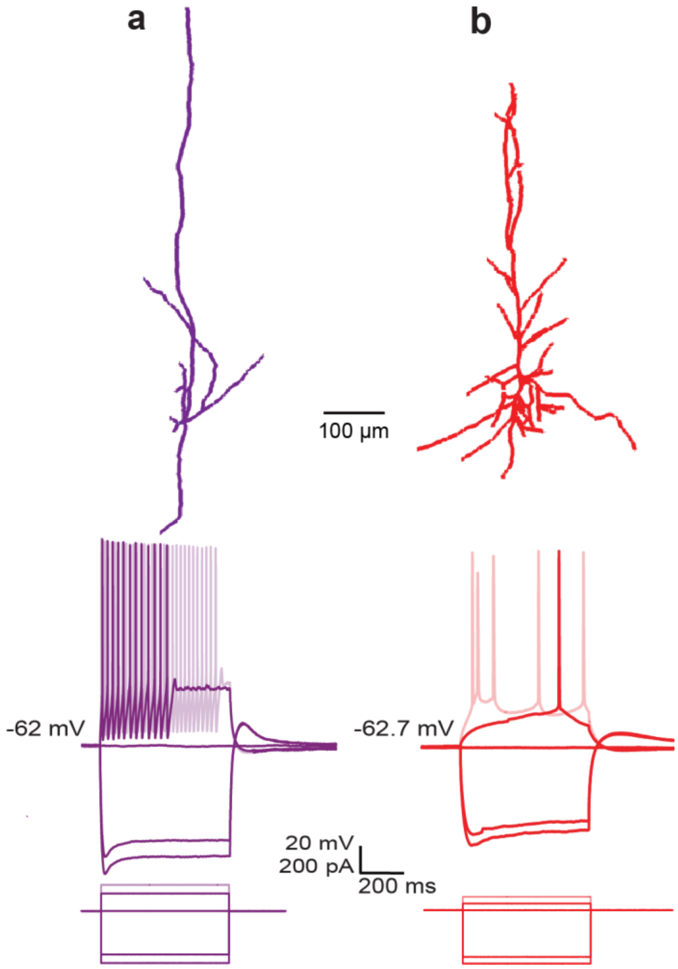

Supplementary Figure 8: Morphological evidence for interneuron sampling. **a** Morphological reconstruction and firing pattern following hyperpolarizing and depolarizing current injections for a putative interneuron; 2100 µm below pia. **b** Morphological reconstruction from a confirmed pyramidal cell (cell f in Figure 1) and related firing pattern following hyperpolarizing and depolarizing current injections; 1800 µm below pia.

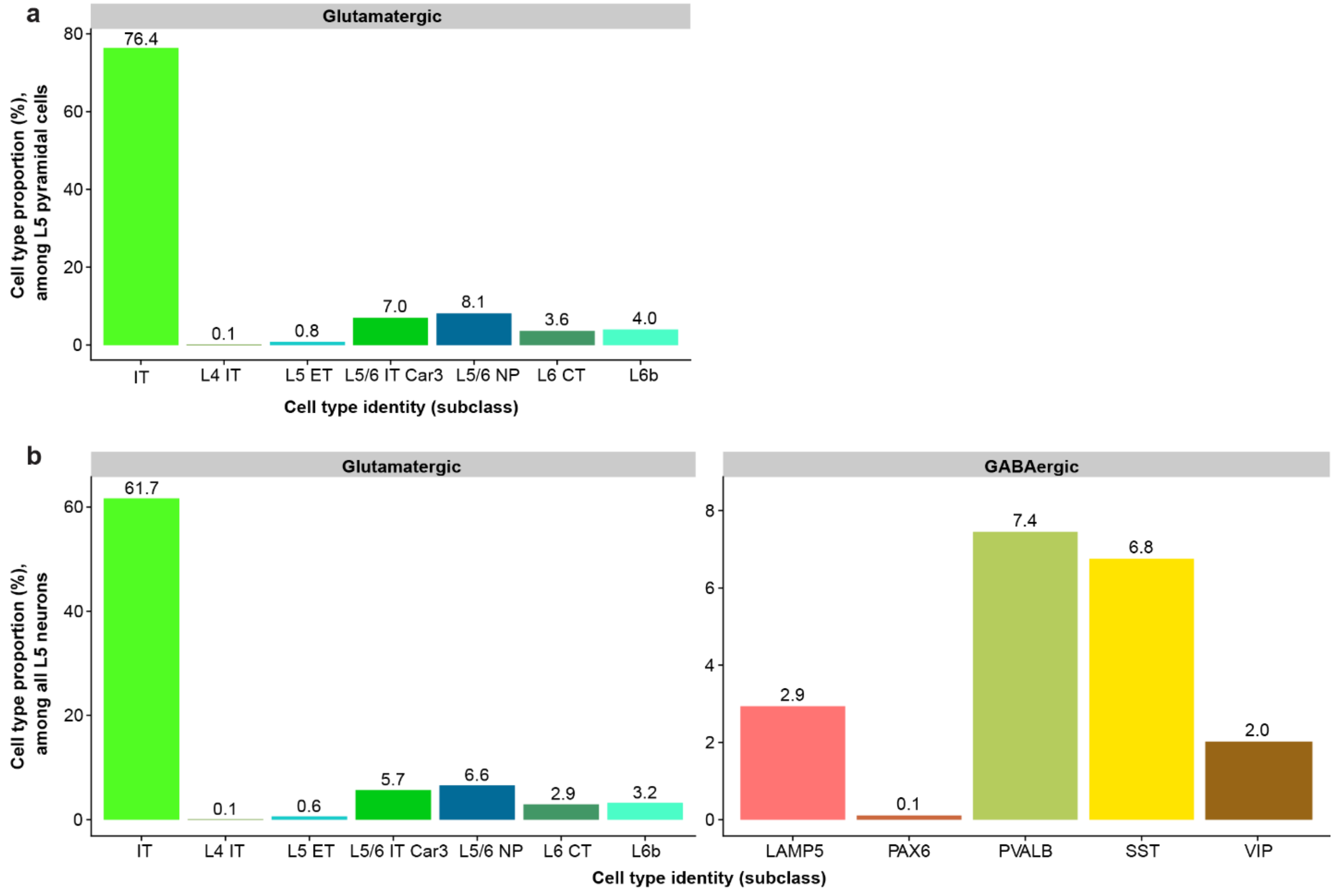

Supplementary Figure 9: Cell type proportions and in human MTG Layer 5 based on single-nucleus RNAseq reference data. Data are replotted from Hodge et.al.[1], based on cortical layer-specific tissue dissections . **a.** Cell type proportions among sampled excitatory (i.e., Glutamatergic) nuclei in L5 (n = 2200 cells total). X-axis denotes cell type labels, annotated to the "subclass" cell type resolution in Hodge et al, 2019. Label "L5 ET" denotes extra-telencephalic projecting cells and "IT" denotes intra-telencephalic projecting cells (identified through transcriptomic similarity and homology to the mouse). **b.** Same as a, but for y-axis denotes cell type proportions relative to all neurons sampled in L5 (n = 2725 cells total). Source data are provided as a Source Data file.

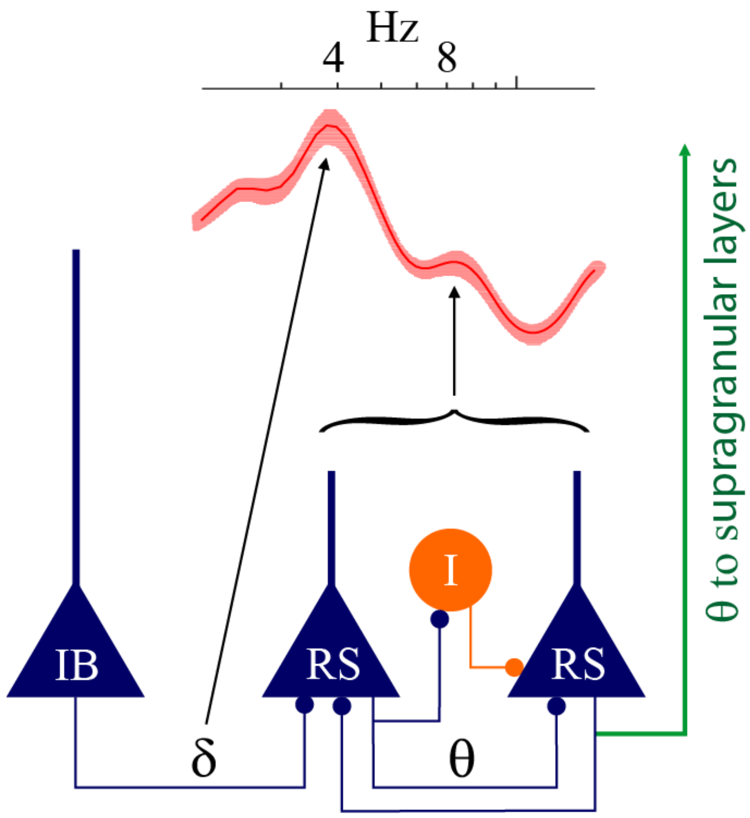

Supplementary Figure 10: Structural circuit motif for L5 theta oscillations. Delta frequency output from intrinsically bursting (IB)[2] neurons is well tracked by regular spiking (RS) neurons that have a peak in G(f) (red) (see Figure 5b in main text) within the delta frequency range. In contrast with IB neurons, RS neurons are poorly adapting, have steep f-I curves, and low rheobase discharge at theta frequency[2]. RS cells drive local circuits at theta frequency range including other RS neurons that track theta well, quantified by the peak at ~8Hz in *G(f)*. Interneurons amplify local activity through rebound excitation (I; orange; see Supplementary Figure 7) in human circuits that are predisposed to reverberant activity[3].

Supplementary references

1 Hodge, R. D. *et al.* Conserved cell types with divergent features in human versus mouse cortex. *Nature* **573**, 61-68 (2019).

2 Carracedo, L. M. *et al.* A neocortical delta rhythm facilitates reciprocal interlaminar interactions via nested theta rhythms. *Journal of Neuroscience* **33**, 10750-10761 (2013).

3 Molnár, G. *et al.* Complex events initiated by individual spikes in the human cerebral cortex. *PLoS biology* **6**, e222 (2008).
