# Supplementary material for: Diversity amongst human cortical pyramidal neurons revealed via their sag currents and frequency preferences
Source: Nat Commun. 2021 May 3;12:2497. doi: 10.1038/s41467-021-22741-9 (PMC8093195; doi:10.1038/s41467-021-22741-9)
Supplement: Supplementary file 3 — Reporting Summary [file 41467_2021_22741_MOESM3_ESM.pdf]

nature research

Corresponding author(s): Taufik. A Valiante

Last updated by author(s): Feb 18, 2021

Reporting Summary

Nature Research wishes to improve the reproducibility of the work that we publish. This form provides structure for consistency and transparency in reporting. For further information on Nature Research policies, see our Editorial Policies and the Editorial Policy Checklist.

Statistics

For all statistical analyses, confirm that the following items are present in the figure legend, table legend, main text, or Methods section.

n/a	Confirmed	
☐	☒	The exact sample size (*n*) for each experimental group/condition, given as a discrete number and unit of measurement
☐	☒	A statement on whether measurements were taken from distinct samples or whether the same sample was measured repeatedly
☐	☒	The statistical test(s) used AND whether they are one- or two-sided<br>*Only common tests should be described solely by name; describe more complex techniques in the Methods section.*
☐	☒	A description of all covariates tested
☐	☒	A description of any assumptions or corrections, such as tests of normality and adjustment for multiple comparisons
☐	☒	A full description of the statistical parameters including central tendency (e.g. means) or other basic estimates (e.g. regression coefficient) AND variation (e.g. standard deviation) or associated estimates of uncertainty (e.g. confidence intervals)
☐	☒	For null hypothesis testing, the test statistic (e.g. *F*, *t*, *r*) with confidence intervals, effect sizes, degrees of freedom and *P* value noted<br>*Give P values as exact values whenever suitable.*
☒	☐	For Bayesian analysis, information on the choice of priors and Markov chain Monte Carlo settings
☐	☒	For hierarchical and complex designs, identification of the appropriate level for tests and full reporting of outcomes
☐	☒	Estimates of effect sizes (e.g. Cohen's *d*, Pearson's *r*), indicating how they were calculated

Our web collection on statistics for biologists contains articles on many of the points above.

Software and code

Policy information about availability of computer code

Data collection	Whole-cell patch-clamp recordings were obtained using a Multiclamp 700A amplifier, Axopatch 200B amplifier, pClamp 9.2 and pClamp 10.6 data acquisition software (Axon instruments, Molecular Devices, USA). Subsequently, electrical signals were digitized at 20 kHz using a 1320X digitizer.
Data analysis	Data were analysed off-line using Clampfit 10.7, Python, MATLAB, and R software.

For manuscripts utilizing custom algorithms or software that are central to the research but not yet described in published literature, software must be made available to editors and reviewers. We strongly encourage code deposition in a community repository (e.g. GitHub). See the Nature Research guidelines for submitting code & software for further information.

Data

Policy information about availability of data

All manuscripts must include a data availability statement. This statement should provide the following information, where applicable:

- Accession codes, unique identifiers, or web links for publicly available datasets
- A list of figures that have associated raw data
- A description of any restrictions on data availability

Data Availability:
Source data are provided with this paper in the Supplementary Materials.
Code Availability:
Computational code for custom analyses are available at the following GitHub repositories: https://github.com/stripathy/valiante_ih (R) and https://github.com/stripathy/valiante_lab_abf_process (Python).

Field-specific reporting

Please select the one below that is the best fit for your research. If you are not sure, read the appropriate sections before making your selection.

- [x] Life sciences
- [ ] Behavioural & social sciences
- [ ] Ecological, evolutionary & environmental sciences

For a reference copy of the document with all sections, see nature.com/documents/nr-reporting-summary-flat.pdf

Life sciences study design

All studies must disclose on these points even when the disclosure is negative.

Sample size	All sample sizes are provided within the text. No explicit pre-calculation of sample sizes needed to establish effects were done prior to conducting this study.
Data exclusions	Descriptions of how data points were excluded are provided in the text.
Replication	We used an external dataset provided by the Allen Institute for Brain Sciences to replication our main findings.
Randomization	Not applicable
Blinding	No explicit blinding strategy was taken here.

Reporting for specific materials, systems and methods

We require information from authors about some types of materials, experimental systems and methods used in many studies. Here, indicate whether each material, system or method listed is relevant to your study. If you are not sure if a list item applies to your research, read the appropriate section before selecting a response.

Materials & experimental systems

n/a	Involved in the study	
☐	☒	Antibodies
☒	☐	Eukaryotic cell lines
☒	☐	Palaeontology and archaeology
☒	☐	Animals and other organisms
☐	☒	Human research participants
☒	☐	Clinical data
☒	☐	Dual use research of concern

Methods

n/a	Involved in the study	
☒	☐	ChIP-seq
☒	☐	Flow cytometry
☒	☐	MRI-based neuroimaging

Antibodies

Antibodies used	streptavidin-conjugated Alexa Fluor 56894; Ca# S32356
Validation	Antibodies used are described in the Methods.

Human research participants

Policy information about studies involving human research participants

Population characteristics	A table providing the characteristics of the population is provided within the manuscript main text.
Recruitment	Patients with intractable epilepsy and brain tumor are selected for this study. Characterization of patients, environmental factor and results of screening examination are not applied in this study. Written informed consent is obtained from all study participants as stated in the research protocol and participants can quit from study any time before surgery time.
Ethics oversight	University Health Network Resaerch Ethics Board

Note that full information on the approval of the study protocol must also be provided in the manuscript.
